# VINS-MKF: A Tightly-Coupled Multi-Keyframe Visual-Inertial Odometry for Accurate and Robust State Estimation

**DOI:** 10.3390/s18114036

**Published:** 2018-11-19

**Authors:** Chaofan Zhang, Yong Liu, Fan Wang, Yingwei Xia, Wen Zhang

**Affiliations:** 1 Institute of Applied Technology, Hefei Institutes of Physical Science, Chinese Academy of Sciences, Hefei 230031, China; liuyong@aiofm.ac.cn (Y.L.); Wanfan8@mail.ustc.edu.cn (F.W.); xiayw@aiofm.ac.cn (Y.X.); 2 Science Island Branch of Graduate School, University of Science and Technology of China, Hefei 230026, China

**Keywords:** state estimation, visual odometry, visual inertial fusion, multiple fisheye cameras, tightly coupled

## Abstract

State estimation is crucial for robot autonomy, visual odometry (VO) has received significant attention in the robotics field because it can provide accurate state estimation. However, the accuracy and robustness of most existing VO methods are degraded in complex conditions, due to the limited field of view (FOV) of the utilized camera. In this paper, we present a novel tightly-coupled multi-keyframe visual-inertial odometry (called VINS-MKF), which can provide an accurate and robust state estimation for robots in an indoor environment. We first modify the monocular ORBSLAM (Oriented FAST and Rotated BRIEF Simultaneous Localization and Mapping) to multiple fisheye cameras alongside an inertial measurement unit (IMU) to provide large FOV visual-inertial information. Then, a novel VO framework is proposed to ensure the efficiency of state estimation, by adopting a GPU (Graphics Processing Unit) based feature extraction method and parallelizing the feature extraction thread that is separated from the tracking thread with the mapping thread. Finally, a nonlinear optimization method is formulated for accurate state estimation, which is characterized as being multi-keyframe, tightly-coupled and visual-inertial. In addition, accurate initialization and a novel MultiCol-IMU camera model are coupled to further improve the performance of VINS-MKF. To the best of our knowledge, it’s the first tightly-coupled multi-keyframe visual-inertial odometry that joins measurements from multiple fisheye cameras and IMU. The performance of the VINS-MKF was validated by extensive experiments using home-made datasets, and it showed improved accuracy and robustness over the state-of-art VINS-Mono.

## 1. Introduction.

Effectively estimating the state of mobile robotic is the basis to ensure their fundamental autonomous capability. Visual odometry (VO) is a well-known technology that uses a camera to estimate mobile robots’ state, and has got significant attention and applications in the robotic field [1]. In general, the performance of VO to estimate the mobile robot’s state depends on the observed environment information by cameras. The accuracy and robustness of VO will degrade if the observed features are insufficient or poor-quality. For example, most existing VO uses a single camera [2,3,4,5] or stereo cameras [6,7,8], their performances are hindered by the limited field of view (FOV) in difficult indoor environments. Besides, VO methods that only depend on visual cues are prone to drift in rapid motion condition as the motion would jolt the cameras. Further improving the performance of the VO is getting plenty of attention in robotics departments and has become a hot area of research for several years [9,10,11].

Recently, we see two growing trends of improving the VO performance: Using multiple cameras (multi-camera VO) [12,13,14,15,16,17,18] and using the inertial measurement unit (IMU) (VIO) [11,19,20,21,22,23,24,25]. Larger FOV given out by multi-camera can provide abundant environment information and rich visual features so that the performance of VO in challenging conditions could be greatly improved. In spite of this, the estimation performance of VO is easily affected by motion ambiguity problem. IMU can provide precise motion information with high frequency and make up for the gap between visual tracking loss. Thus, fusing IMU data to compensate the visual degradation has become more and more popular under challenging conditions, such as rapid motion, strong illumination changes and the FOV contains large moving objects, etc. However, all the benefits of above two methods come at a price: The efficiency and real-time performance of VO is reduced due to the vast data provided by multi-camera and IMU, thus tightly coupling multi-camera and IMU for state estimation is a challenging problem.

**Figure 1:**
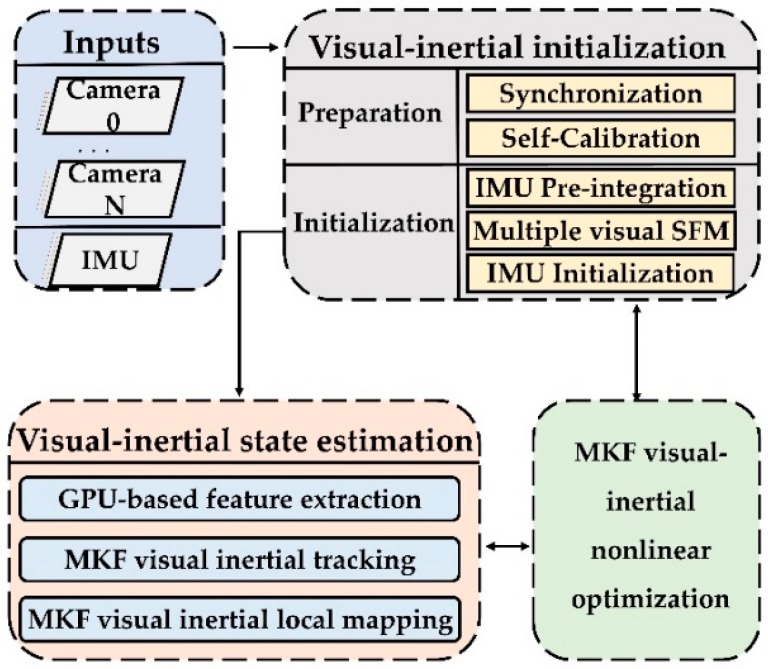
The framework of the proposed VINS-MKF.

Motivated by the above two tendency, we in this work present a novel VO method (VINS-MKF): A tightly-coupled multi-keyframe visual-inertial odometry for accurate and robust state estimation, which is modified from the state-of-art keyframe based monocular ORBSLAM [26] and promoted to provide accurate and robust state estimation for mobile robots in challenging indoor environment. We use multiple fisheye cameras and the IMU to modify the ORBSLAM for providing abundant environment information. The efficiency cost problem caused by the vast information was addressed, by adopting a GPU accelerated feature extraction method and separating the feature extraction from the tracking thread and parallelized with the mapping thread. Furthermore, a nonlinear optimization method is formulated to further ensure the performance of state estimation, which is characterized as being multi-keyframe, tightly-coupled and visual-inertial. In addition, three novel tips, including accurate initialization with a hardware synchronization mechanism and a self-calibration method, a MultiCol-IMU camera model, and an improved multi-keyframe double window structure, are coupled to the VINS-MKF to improve the performance of the state estimation. The framework of the proposed VINS-MKF is shown as Figure 1. Our main contributions are as follows:

- For higher accurate and robust VO state estimation, a hyper graph structure based nonlinear optimization was formulated, which characterized by multi-keyframe, tightly-coupled and visual-inertial combination.
- To estimate the state of mobile robot efficiently, a novel VO state estimation framework was proposed, in which a GPU based feature extraction thread was parallelized with tracking and mapping thread.
- To further ensure the precision of state estimation, a novel MultiCol-IMU camera model and an accurate initialization method with a hardware synchronization mechanism and self-calibration method were presented.
- The performance of the VINS-MKF was validated by tremendous experiments on home-made datasets, and the improved accuracy and robustness was demonstrated by comparing against the state-of-the-art VINS-Mono algorithm.
- To the best of our knowledge, the proposed VINS-MKF is the first tightly-coupled multi-keyframe visual-inertial odometry based on monocular ORBSLAM, modified with multiple fisheye cameras alongside an inertial measurement unit (IMU).

The rest of this paper is organized as follows. In Section 2, we briefly discuss the relevant work. Then, the essential aspect of state estimation, i.e., the proposed multi-keyframe tightly-coupled visual-inertial nonlinear optimization, is introduced in Section 3. In Section 4, we describe the visual-inertial state estimation. Section 5 shows the experiments details and results. Finally, we conclude the paper and discuss future research in Section 6.

## 2. Related Work

From the technical point of views, VSLAM and VO are highly relevant techniques because both techniques basically estimate sensor positions [27]. Over the past few decades, various VO and VSLAM methods have been proposed, in this section, we briefly review the VO and VSLAM methods that most relevant to this paper.

VO can be categorized into two predominant groups according to the processing technique: Filtering method [20,28,29,30,31] and keyframe based method [3,9,10,26,32,33,34,35,36]. Keyframe based method provides better accuracy for the same computational work than filtering method. Further details of the two approaches and some relative advantages and disadvantages can be found in Reference [37]. Among plenty keyframe based scholarly works, PTAM [32] is the first real-time SLAM (Simultaneous Localization and Mapping) system that was conducted for performing optimization over keyframes. It seminally splits tracking and mapping into two parallelized threads, this model has become a standard paradigm for subsequent VO algorithms. Later, Strasdat et al. in Reference [35] introduced the double window approach and the co-visibility concept for optimizing, selecting and constraining keyframes. Recently, Raul et al. extended the PTAM by adding a loop closure thread and the relocalization function and presented the state-of-art keyframe based monocular ORBSLAM [26], which have better accuracy and robustness than PTAM and can be performed in real-time in various environments.

With the advancement of computer vision technology and robotics community, there have been increasing tendency of using the multi-camera VO for state estimation in robotics field. Early multi-camera works mainly focus on the structure from motion (SFM) [12,15,38], recently, lots of multi-camera pose estimation works for mobile robots have been proposed [14,16,17,18,38,39,40,41]. In Reference [16], Harmat et al. presented MCPTAM and investigated the influence of different camera layout structure on the positioning accuracy of UAV and introduced the multi-keyframe to the modified PTAM in Reference [39]. Similar to our work, MCPTAM uses multiple fisheye cameras, and it takes advantage of the generic polynomial model, i.e., the Taylor omnidirectional camera model. Different from our work, the mapping thread of MCPTAM is the same as the original PTAM, while we use double window optimization for mapping. Later, Heng et al. presented a multi-camera work in Reference [14] and coupled four cameras rigidly, with pairs of cameras being paired in stereo configurations. This work had a similar mapping pipeline with ORB-SLAM. Recently, Urban et al. used a hyper-graph based MultiCol model to extend the ORBSLAM and presented the MultiCol-SLAM [18], which is applicable to arbitrary, rigidly coupled multi-camera system. This work is very similar to our work, while it still has the same framework as ORBSLAM. 

Visual-inertial Odometry (VIO) can be categorized into two types: Tightly-coupled [11,22,23,24,25] and loosely-coupled [21,42,43]. Tightly-coupled VIO can optimize the data from the visual and inertial sensor in order to assure the results’ accuracy. While loosely-coupled VIO performs state estimation by two separate estimators and leads to sub-optimal results. Recent visual inertial odometry studies are focused on tightly-coupled VIO. In Reference [11], Leutenegger et al. adopted a nonlinear optimization method over the tightly coupled visual and inertial cost terms and presented a keyframe based VIO. But its marginalization mechanism that dropping the marginalized landmarks from the system causes the approach to be sub-optimal. Forster et al. proposed an on-manifold based pre-integration technique for VIO state estimation in Reference [23]. Later, by enabling loop closure and the previously estimated 3D maps to be reused, Raul et al. presented the ORB-VISLAM in Reference [24], which was a real-time tightly-coupled monocular visual-inertial SLAM system, however it is not available to the public. Recently, Qin et al. developed the most popular VINS-Mono algorithm in Reference [25], they employed point features to optimize IMU body states and performed nonlinear optimization in a sliding window. However, the utilized optical flow feature extraction method and its growing accumulative errors limit its accuracy. None of the above visual inertial fusion studies have considered multiple cameras.

The most relevant work with this paper is the work in Reference [44], Houben et al. extended the monocular ORB-SLAM with multiple cameras alongside an IMU and presented a multi-camera visual inertial work for micro aerial vehicles. However, the work adopted the loosely-coupling method and only aggregated IMU readings to one motion prior. Besides, it is limited to one camera that is directed towards the axis of rotation. Different to that work, in this paper, we adopt tightly-coupled fusion methods and we have no limitations with regard to the camera direction.

For the purpose of an accurate and robust state estimation, the presented VINS-MKF tightly-coupled multi-keyframe visual measurements and IMU motion measurements for state estimation, it’s based on the framework of ORBSLAM and it has some essential modifications and improvements.

## 3. Multi-Keyframe Tightly-Coupled Visual-Inertial Nonlinear Optimization

For the proposed VINS-MKF, the state estimation problem is equivalent to the Maximum a posterior probability (MAP) of the given visual-inertial measurements [45], we sought to formulate a multi-keyframe tightly-coupled visual-inertial nonlinear optimization method, to gain better state estimation accuracy and reduce errors caused by sensor noise and modelling error. In the following, we will detail the MultiCol-IMU model and IMU pre-integration, along with the derivation and solution of the proposed nonlinear optimization.

### 3.1. MultiCol-IMU Camera Model and Structure

**Figure 2:**
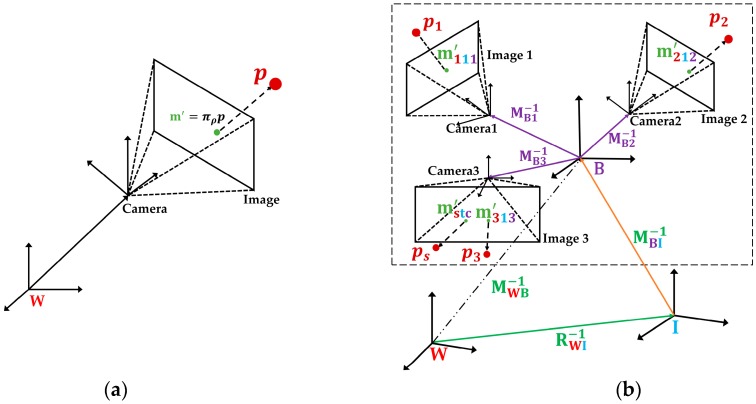
Different camera models. (**a**) Camera model for a single camera; (**b**) The proposed Multicol-IMU camera model. Compared to the single camera model in Figure 2a, the proposed MultiCol-IMU camera model in Figure 2b had two intermediate frames: A body frame **B** and an IMU frame **I**.

As we extend the ORBSLAM [26] with multiple fisheye cameras and an IMU unit, the pinhole camera model in Reference [39] are not suitable for the proposed VINS-MKF. Thus, inspired by the works in Reference [18], we propose a MultiCol-IMU camera model to model the multiple fisheye cameras. Figure 2b shows the proposed MultiCol-IMU camera model and its structure. We briefly discuss how to describe the relationship between a point on the image plane and its corresponding world point. 

#### 3.1.1. Camera Model for Single Camera 

As shown in Figure 2a, for a point $m={\left[u,v\right]}^{T}$ in camera coordinate system, we gain its corresponding image point $m\prime ={\left[u^{\prime },v^{\prime }\right]}^{T}$ through an affine transformation $m\prime =Am+Q_{c}$, $Q_{c}={\left[o_{u},o_{v}\right]}^{T}$ is the optic axis offset. This process can be described as:

(1) $$\;\left[\begin{array}{c} u^{\prime }\\ v^{\prime } \end{array}\right]=\left[\begin{array}{cc} c & d\\ e & 1 \end{array}\right]\left[\begin{array}{c} u\\ v \end{array}\right]+\left[\begin{array}{c} o_{u}\\ o_{v} \end{array}\right]\;$$

Then, we gain the corresponding point p in the world coordinate system through the imaging projection function g. This process is described in (2). D is the exterior orientation parameter and λ is the depth scale factor. Function f(ρ) represents the optical surface characteristics, and ρ is the Euclidean distance from the image center. Function f(ρ) has various forms; we use the polynomial model of f(ρ), since it is more suitable for the fisheye cameras and has a better accuracy.

(2) $$\lambda g\left(m\right)=\lambda \left[\begin{array}{c} u\\ v\\ f\left(u,v\right) \end{array}\right]=\lambda \left[\begin{array}{c} u\\ v\\ f\left(\rho \right) \end{array}\right]=D\left[\begin{array}{c} \begin{array}{c} X\\ Y \end{array}\\ \begin{array}{c} Z\\ 1 \end{array} \end{array}\right],\;\rho =\sqrt{u^{2}+v^{2}},\lambda >0\;$$

(3) $$f\left(\rho \right)=a_{0}+a_{2}\rho^{2}+\ldots .\;+\;a_{n}\rho^{n}.$$

We use $\pi_{\rho }$ to describes the camera model, i.e., the above two processes that map a 3D scene points to its location on the 2D image plane.

#### 3.1.2. MultiCol-IMU Camera Model

As shown in Figure 2b, the proposed MultiCol-IMU camera model had two intermediate frames: The body frame **B** and the IMU frame **I**. Since we used multiple fisheye cameras, multi-camera observations of the scene points existed at the same time t, and all the observations had to be embedded into the observation equations. Inspired by Reference [18], we used an intermediate frame, the body frame **B**, to represent the absolute pose of the proposed VINS-MKF system. The body frame **B** allowed for separating of the observations from each camera, and for combing all observations to one observation equation simultaneously. Besides, we added the IMU frame **I** to the transformation between world frame and the body frame, in order to unify the coordinate systems and to facilitate the following calculation. 

Given: s=1,2,…S represents the scene points, and c=1,2…C represents camera c in MCS, t=1,2…T represents the pose of MCS at time t. Thus, by using the proposed MultiCol-IMU camera model, the projection of transforming 3D points in the world reference into 2D points on the image can be mathematically described as:

(4) mstc'=πρ(pstc)=πρ(MBC−1MIB−1RWI−1(ps−pIW).

### 3.2. IMU Pre-Integration

Generally, IMU pre-integration [46] is necessary, as inertial measurements come at a high rate and real-time optimization becomes infeasible as the trajectory grows over time and the number of variables grows rapidly. In this work, we used the on-manifold pre-integration theory in Reference [47] to pre-integrate IMU measurements. A brief description is given here, and more details can be found in Reference [47]. 

IMU motion model can be described by the Equation (5):

(5) RWIt+1=RWItEXP(∫T∈(t,t+1)(ωIT−bgT−ηgT)dT),PIt+1W=PItW+∫T∈(t,t+1)(VItW+∫T∈(t,t+1)(RWIT(aIT−baT−ηaT)−gw)dT)dT,VIt+1W=VItW+∫T∈(t,t+1)(RWIT(aIT−baT−ηaT)−gW)dT

R, P and V are respectively, the rotation, position and velocity of the IMU, the instantaneous angular velocity $\omega_{I}$ and accelerator $a_{I}$ are derived from the measurements of the IMU. We gained the estimation of the motion between time t+1 and t from Equation (5), but it has a drawback in that the integration in (5) has to be repeated whenever the linearization point at time t changed. Thus, we changed the reference coordinate to solve the weakness and to gain the motion increments that were only dependent on ω and a, independent of the pose and velocity at t, as Equation (6) shows:

(6) (RWIt)−1RWIt+1=EXP(∫T∈(t,t+1)(ωIT−bWT−ηWT)dT)=ΔRtt+1,(RWIt)−1(PIt+1W−PItW−VItWΔt−12gWΔt2)=∬T∈(t,t+1)(RWIt)−1RWIT(aIT−baT−ηaT)(dT)2=ΔPtt+1,(RWIt)−1(VIt+1W−VItW−gWΔt)=∫T∈(t,t+1)(RWIt)−1RWIT(aIT−baT−ηaT)dT=ΔVtt+1

$\Delta R_{t}^{t+1}$, $\Delta P_{t}^{t+1}$ and $\Delta V_{t}^{t+1}$ are the changes of R, P, and V, respectively, from t to t+1 at the IMU coordinate at time t, affected by the real $\omega_{B}$ and $a_{B}$. Through Equation (6), we associated the IMU state with the measurements. Next, we isolated the noise terms of the individual inertial measurements in (6), and from the following Taylor first-order approximation, we can get:

(7) $$\Delta R_{t}^{t+1}=\Delta {\tilde{R}}_{t}^{t+1}EXP\left(\delta \phi_{t}^{t+1}\right)\approx \Delta {\bar{R}}_{t}^{t+1}EXP\left(J_{w}^{R}\delta b_{w}^{t}\right)EXP(\phi_{t}^{t+1}),\;\Delta P_{t}^{t+1}=\Delta {\tilde{P}}_{t}^{t+1}+\delta P_{t}^{t+1}\approx \Delta {\bar{P}}_{t}^{t+1}+J_{w}^{P}\delta b_{w}^{t}+J_{a}^{P}\delta b_{a}^{t}+\delta P_{t}^{t+1},\;\Delta V_{t}^{t+1}=\Delta {\tilde{V}}_{t}^{t+1}+\delta V_{t}^{t+1}\approx \Delta {\bar{V}}_{t}^{t+1}+J_{w}^{V}\delta b_{w}^{t}+J_{a}^{V}\delta b_{a}^{t}+\delta V_{t}^{t+1}.$$

$\Delta {\tilde{R}}_{t}^{t+1}$, $\Delta {\tilde{P}}_{t}^{t+1}$ and $\Delta {\tilde{V}}_{t}^{t+1}$ stand for the condition that $\Delta R_{t}^{t+1}$, $\Delta P_{t}^{t+1}$ and $\Delta V_{t}^{t+1}$ are affected by random noises, $\Delta {\bar{R}}_{t}^{t+1}$, $\Delta {\bar{P}}_{t}^{t+1}$ and $\Delta {\bar{V}}_{t}^{t+1}$ mean that there is no bias changes in $\Delta {\tilde{R}}_{t}^{t+1}$, $\Delta {\tilde{P}}_{t}^{t+1}$, and $\Delta {\tilde{V}}_{t}^{t+1}$ respectively. The Jacobian matrix J is a first-order approximation of the effect of changing the biases b. When IMU measurements arrive, we can efficiently compute both the pre-integrations and the Jacobians iteratively. $\delta b^{t}$ is the small perturbation of bias. Assuming the measurement error was zero, we gained the IMU measurement model:

(8) ΔR˜tt+1=(RWIt)−1RWIt+1,ΔP˜tt+1=(RWIt)−1(PIt+1W−PItW−VItWΔt−12gWΔt2),ΔV˜tt+1=(RWIt)−1(VIt+1W−VItW−gWΔt).

### 3.3. Derivation of the Proposed Nonlinear Optimization 

We sought to formulate a tightly-coupled multi-keyframe nonlinear optimization for the highly accurate and robust estimate of system states and landmark positions, using both multi-keyframe visual measurements and IMU inertial measurement. The graph representation is shown in Section 4.3.1. In this section, we briefly describe the derivation process of the proposed nonlinear optimization method. 

#### 3.3.1. The States 

Given that Xt=(RWIt,PItW,VItW,bwt,bat) denotes the system state when landmarks l are seen at t, thus, the variables to be estimated of our multi-keyframe tightly-coupled VIO can be described as: χ=(RWI,PIW,VIW,bw,ba).

#### 3.3.2. The Measurements 

We defined the input measurements $Z=\left\{Z_{I},Z_{C}\right\}$, where $Z_{C}$ and $Z_{I}$ are image measurements and IMU measurements respectively. We denoted the image measurements as $Z_{C}$, and $Z_{stc}$ stands for the image measurements when landmarks s are seen at t.

We denoted $Z_{I}$ as the IMU measurements, $Z_{It}^{t+1}$ are the IMU measurements between two consecutive keyframes, t and t+1. Depending on the IMU measurement rate and the frequency of selected keyframes, each set $Z_{It}^{t+1}$ can contain from a small number of IMU measurements, to hundreds of IMU measurements.

#### 3.3.3. Derivation of the Nonlinear Optimization 

The purpose of the proposed nonlinear optimization is to estimate the system states χ accurately when given measurements Z. Since it is difficult to obtain the exact system states because of the existence of noises, we defined the state estimation problem as a conditional probability distribution p(χ|Z); according to the Bayesian principle, we obtained Equation (9), p(χ) are the priors. Since the state estimation only depended on the system measurements Z, the state estimation problem amounted to solving the maximum likelihood estimation $\chi^{*}$, i.e., to find which state estimate is most likely to present the current observations $\chi^{*}$, this can be expressed with Equation (10):

(9) p(χ|Z)∝p(χ)p(Z|χ),

(10) $$\chi^{*}=\mathrm{argmax}\;p\left(\chi \right)p\left(Z|\chi \right)=\;\mathrm{argmax}\;p\left(\chi \right)\prod_{t}P(Z_{It}^{t+1}|X_{t},X_{t+1})\prod_{s}\prod_{t}\prod_{c=1,2,3}P(Z_{sct}|X_{t}).$$

Through mathematical analysis, the MAP estimate $\chi^{*}$, which is equivalent to the minimum of the negative log-posterior, we assume that the noises obey the zero-mean Gaussian noise, and the negative log-posterior can be written as a sum of squared residual errors:

(11) $$\begin{array}{cc} \chi^{*}=\mathrm{argmin}- & \log_{e}\;p\left(\chi \right)p\left(Z|\chi \right)\\ & =\mathrm{argmin}\;\{\|e_{prior}{\|}_{\Sigma_{0}}^{2}+\sum_{t}\|e{\left(Z_{It}^{t+1})\right\|}_{\Sigma_{t}^{t+1}}^{2}\\ & +\sum_{s}\sum_{t}\sum_{c=1,2,3}H\left(\|e{\left(Z_{stc})\right\|}_{\Sigma_{stc}}^{2}\right)\} \end{array}$$

$\|e_{prior}{\|}_{\sum_{0}}^{2}$ is the prior of system state residuals, $e\left(Z_{It}^{t+1}\right)$ and $e\left(Z_{stc}\right)$ are the residual errors associated to the measurements, $\sum_{t}^{t+1}$, $\sum_{stc}$ are the corresponding covariance matrices. H(⋅) is the Huber norm. Roughly speaking, given the state χ, the residual error is a function of χ that quantifies the mismatch between measurements and its prediction. Next, we use nonlinear method to solve the problem.

### 3.4. The Solution to the Nonlinear Optimization

We used the iteration solution ideas to solve the nonlinear optimization Equation (11), and we used the Levenberg-Marquardt method [48] to provides a more stable and accurate increment Δx. There exists a formulation in the Levenberg-Marquardt method:

(12) $$(H+\lambda D^{T}D)\Delta x=g,\;\left(J{\left(x\right)}^{T}J\left(x\right)+\lambda I\right)\Delta x=-J{\left(x\right)}^{T}f\left(x\right).$$

From Equation (12) we can see that the solution highly depends on the Jacobian matrix, thus the key step of solving nonlinear optimization is to calculate the Jacobian matrix. 

#### 3.4.1. Multi-Keyframe Reprojection Error Term 

Assume that the landmark l is seen at keyframe t, for clarity, we express the camera model in Section 3.1 as (13), then the residual of the multi-keyframe reprojection measurement $\Delta z_{stc}$ can be defined as (14):

(13) zstc=πp(pstc)=πp(MBC−1MIB−1RWI−1(ps−PIW)),

(14) e(zstc)=Zstc−πp(MBC−1MIB−1RWI−1(pi−PIW)).

#### 3.4.2. IMU Error Term 

In Equation (15), we modeled the biases with a “Brownian motion”. By adding the biases residuals to the IMU measurements error term $\sum_{t}\|b^{t+1}-b^{t}{\|}_{\sum_{b}}^{2}$, where $\sum_{b}$ are the corresponding covariance matrices, we gained the IMU error term in Equation (16):

(15) $${\dot{b}}_{\left(t\right)}^{w}=\eta^{bw},\;{\dot{b}}_{\left(t\right)}^{a}=\eta^{ba},$$

(16) e(ZItt+1)=[e(Rtt+1)e(Ptt+1)e(Vtt+1)e(bw)e(ba)]=[loge((ΔR˜tt+1)(RWIt)−1)v(RWIt)−1(PIt+1W−PItW−VIt+1WΔt−12gWΔt2)−ΔP˜tt+1(RWIt)−1(VIt+1W−VItW−gWΔt)−ΔV˜tt+1bWt+1−bWtbat+1−bat]∈R15.

$\Delta {\tilde{R}}_{t+1}^{t}$ is the homogeneous transformation of $\Delta {\tilde{R}}_{t}^{t+1}$.

#### 3.4.3. The Solution to the Nonlinear Optimization 

We first analyzed the Jacobian matrix of the error terms. Taking the IMU measurement model as an example, given the initial value of the state variable to be estimated, similar to Equation (17), we added a perturbation vector δχ (Equation (18)) to the state variable χ, and the Jacobian matrix $J_{I}$ (Equation (19)) can be gained.

(17) RWI→RWIEXP(δϕ) PIW→PIW+RWIδPI VIW→VIW+δVIWδbw→δbw+δb˜w δba→δba+δb˜a

(18) δχ=(δϕ,δPI,δVIW,δb˜w,δb˜a),

(19) $$J_{I}=\left(\begin{array}{c} \begin{array}{c} J_{R}\\ J_{P} \end{array}\\ \begin{array}{c} J_{V}\\ J_{b^{w}} \end{array}\\ J_{b^{a}} \end{array}\right)=(\;\begin{array}{c} \begin{array}{c} \underset{\delta \chi \to 0}{\lim}\frac{\partial e\left(\Delta R\right)}{\partial \delta \chi }\\ \underset{\delta \chi \to 0}{\lim}\frac{\partial e\left(\Delta P\right)}{\partial \delta \chi } \end{array}\\ \begin{array}{c} \underset{\delta \chi \to 0}{\lim}\frac{\partial e\left(\Delta V\right)}{\partial \delta \chi }\\ \underset{\delta \chi \to 0}{\lim}\frac{\partial e\left(\Delta b^{w}\right)}{\partial \delta \chi } \end{array}\\ \underset{\delta \chi \to 0}{\lim}\frac{\partial e\left(\Delta b^{a}\right)}{\partial \delta \chi } \end{array}).$$

Defining $J_{t}^{t+1}$ is the Jacobian matrix of the IMU error term from t to t+1, $J_{stc}$ is the Jacobian matrix of error term when camera c is observing the landmark s at time t. We obtained other Jacobian matrices by the same principle. Thus, the Jacobian J of the whole nonlinear optimization can be gained by Equation (20). According to the Equation (12), we can obtain Δx, and finally we can solve the nonlinear optimization.

(20) $$J^{T}J=\sum_{t}{\left(J_{t}^{t+1}\right)}^{T}J_{t}^{t+1}+\sum_{c}\sum_{t}\sum_{l}{\left(J_{stc}\right)}^{T}J_{stc}\;$$

## 4. Multi-Keyframe Tightly-Coupled Visual-Inertial Odometry

In this section, we describe the basic structure of the proposed VINS-MKF, as shown in Figure 1, and we detail the modifications to the ORBSLAM. The algorithm began with an accurate visual inertial initialization (Section 4.1), which provided all necessary values, including pose, velocity, gravity vector, gyroscope bias, and 3D feature location, for bootstrapping the subsequent visual-inertial state estimation. The visual-inertial state estimation included three parallel processes: GPU based feature extraction (Section 4.2), multi-keyframe visual-inertial tracking (Section 4.3) and multi-keyframe visual-inertial local mapping (Section 4.4). The parallelized GPU-based feature extraction ensures the system efficiency. The tracking and local mapping processes aimed to address the state estimation problem by tightly fusing multi-keyframe visual measurements and IMU information. These three modules ran concurrently in a multi-thread setting.

### 4.1. Visual Inertial Initialization

**Figure 3:**
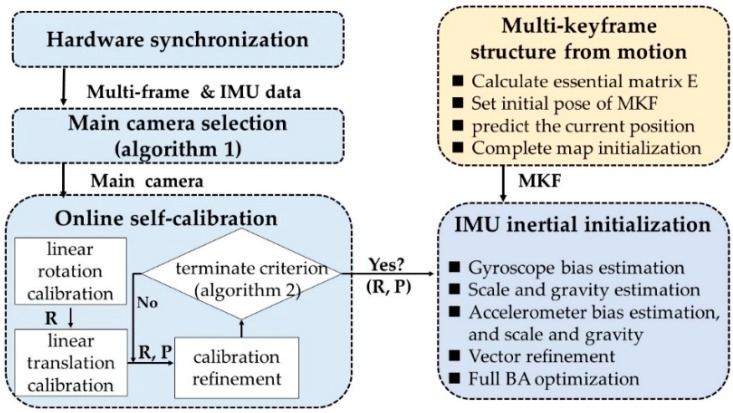
The procedure of visual-inertial initialization. The initialization includes two pre-requires: Hardware synchronization mechanism and online self-calibration. Following the multi-keyframe structure from motion and IMU inertial initialization.

As the proposed VINS-MKF was a highly nonlinear system, good initial values significantly affected the VINS-MKF’s accuracy, thus a robust and accurate initialization was critical for the VINS-MKF. Based on the multi-keyframe measurements, we present a loosely coupled visual-inertial initialization method, as Figure 3 shows. We added a hardware synchronization mechanism to provide sensor clock synchronization, and we introduced a self-calibration method for an accurate camera−IMU external parameters. 

#### 4.1.1. Initialization Pre-Requirements

**Figure 4:**
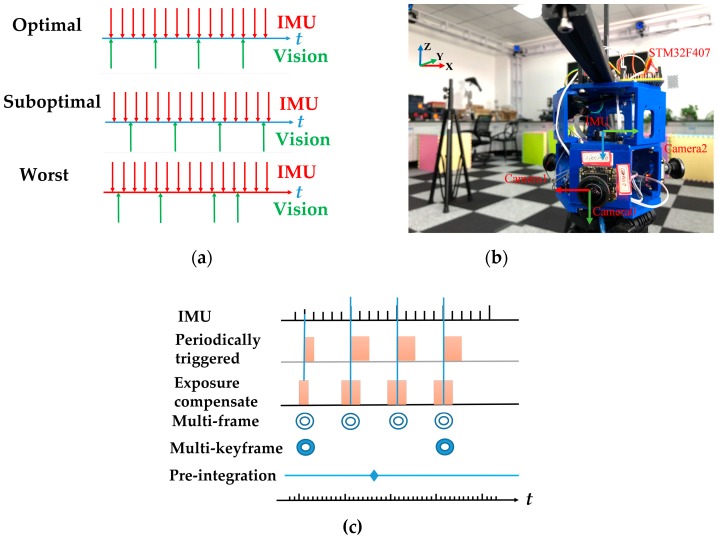
Sensor Synchronization and Data Acquisition. (**a**) Different conditions of synchronization and timestamps of sensors; (**b**) the hardware configuration and synchronization scheme in this work. The STM32F407 was used to ensure the synchronization between IMU and multiple fisheye cameras. The STM32F407 calculates precise (millisecond) timestamps for each IMU measurement (200 Hz). At certain timestamps (20 Hz), it will trigger the multiple fisheye cameras to capture new images (i.e., the IMU triggers the multiple fisheye cameras through STM32F407). According to the method in References [49,50], the accurate timestamp of multiple fisheye cameras will be got through adding half the exposure time to the IMU’s timestamps. (**c**) The principle of hardware synchronization method with exposure compensation.

**Sensor Synchronization and Data Acquisition:** Accurate sensor clock synchronization of multiple cameras and the IMU is important for the tight integration of the visual-inertial system. The different conditions of synchronization and timestamps of sensors are shown in Figure 4a. To achieve optimal conditions, i.e., sensors that are perfectly synchronized, we used a hardware synchronization method with exposure compensation [49,50] to trigger the multi-cameras and the IMU, Figure 4c briefly shows the principle. This work was achieved using STM32F407 (STMicroelectronics, Geneva, Switzerland), the hardware configuration and synchronization scheme in this work can be found in Figure 4b. 

**Online Self-Calibration and Adaptive Main Camera Selection:** An accurate camera-IMU extrinsic parameter is crucial for multi-camera visual-inertial initialization. In this paper, we used the state-of-the-art online self-calibration method proposed in Reference [51] and we made two improvements to calibrate the multiple cameras and the IMU. The procedure began with an adaptive main camera selection mechanism (shown in Algorithm 1) to select a main camera, following three steps to perform external parameter calibration with the IMU. Besides, we introduce an external parameters calibration terminate criterion (shown in Algorithm 2) to ensure yielding accurate camera-IMU external parameters and to launch the multi-camera visual-inertial initialization procedure. The proposed method can automatically estimate precise extrinsic parameters without knowing the mechanical configuration and can be operated in natural environments. The gained orientation (roll, pitch, and yaw) using our method between main camera (take camera 0 as an example) and IMU are [87.679, 179.957, −91.799], and the translation (x, y, and z) are [−0.102, −0.003, 0.040].

**Algorithm 1.** Adaptive selection of the main camera.  th1←0  th2←0  **for** c < numcams **do**  **if** inliers[c] > th1 && translational[c] > th2 **then**  mainCam←c     th1←inliers[c]  th2←translational[c]  **end if**  **end for**

**Algorithm 2.** The external parameters calibration terminate criterion.**Input:** the local period *t*, time increment *∆t*, convergence time *T*, the standard deviations $\sigma_{\mathrm{oy}}$, $\sigma_{\mathrm{op}}$, $\sigma_{\mathrm{or}},\sigma_{\mathrm{tx}}$, $\sigma_{\mathrm{ty}}$, $\sigma_{\mathrm{tz}}$ of the six axes (yaw, pitch, roll, x, y, z), threshold value *∆*σ. **Output:** the external parameters *R, P* **Process:**   *T*
← 0;   while *T* < 60 do   update (R, P) with the most recent convergence value   **if**
*t* then  if $\sigma_{\mathrm{oy}}$ < *∆*σ, $\sigma_{\mathrm{op}}$ < *∆*σ, $\sigma_{\mathrm{or}}$ < *∆*$\sigma ,\sigma_{\mathrm{tx}}$ < *∆*σ, $\sigma_{\mathrm{ty}}$ < *∆*σ, $\sigma_{\mathrm{tz}}$< *∆*σ
**then**     break;     **end if**    **end if**    *T* ← *T* + *∆t*;   **end**

#### 4.1.2. Multi-Keyframe SFM

We adopted a loosely-coupled sensor fusion method to get initial values, as Structure from Motion (SFM) has a good property of initialization. The procedure can be seen from Figure 3. Once the external parameters calibration terminate criterion were accepted, the multi-camera visual-Inertial initialization procedure began to perform the multi-keyframe SFM. Different to ORBSLAM, in our work, we used a practical way to complete the multi-frame SFM. After building a multi-frame, the essential matrix E in a RANSAC loop between the same camera from different MCS poses is estimated. Then the main camera’s pose was regarded as the initial pose of multi-frame; and the current position of the system was predicted by calculating the relative orientation between the last two multi-frames. Finally, the map initialization was completed and all variables were observable, by using pure multi-visual information. It should be noted that the map initialization method proposed in the original ORBSLAM had several limitations for our method. Such as, the camera matrix did not exist, due to the employed fisheye cameras and the MultiCol-IMU camera model, thus we could not calculate both F and H matrices that contained the perspective camera matrix K.

Note that in this paper, although we used multiple cameras, these cameras were nonoverlapping, the scale could not be observed directly.

#### 4.1.3. IMU Inertial Initialization

The final step of visual-inertial initialization was the IMU inertial initialization. We use the method proposed in Reference [24], which simultaneously estimated the gyroscope and accelerometer bias during the initialization phase. We made some improvements to the initialization method [24] to suitable for our VINS-MKF, we used the pose of multi-camera instead of the pose of single camera to acquire the parameters, which includes the visual scale, the gravity, the biases of gyroscope and accelerometer, and velocity, the steps are shown in Figure 3.

### 4.2. GPU Based Feature Extraction 

**Figure 5:**
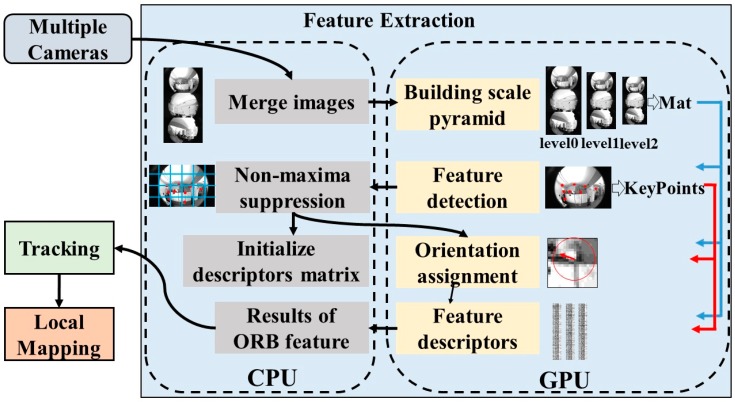
The framework of GPU based feature extraction. The CPU-GPU combination optimization strategy and the parallelization steps of feature extraction are shown in this figure.

Feature detection is an important, and time-consuming step, for the proposed VINS-MKF. The system can hardly meet the real-time calculation requirements for low-power embedded systems with low CPU (Central Processing Units) computational efficiencies, due to the vast multi-camera visual-inertial information. To address this problem, in this paper, a CPU-GPU combination optimization strategy was used to accelerate the feature extraction algorithm, and the feature extraction was separated from tracking module and executed as an independent thread by the GPU to take full advantage of the CPU and GPU computing resources, as Figure 5 shows.

As we can see from Figure 5, the CUDA was used to implement the feature extraction algorithm in parallel on the GPU, which had the advantage that our feature extraction algorithm can be executed on any CUDA-supported GPU. The parallelization steps of feature extraction are also shown in Figure 5. On the other hand, we noted that feature extraction was just part of tracking module, the GPU was always free while other operations were executed in the tracking module, and they were only reused after the calculation of tracking module is completed and after new images were read. Thus, on the CPU, we separated the feature extraction algorithm from tracking, and we ran feature extraction, tracking and local mapping in parallel to further improve the efficiency of VINS-MKF.

Note that before using the CPU-GPU combination optimization strategy to accelerate the feature extraction, we performed some improvements to modify the ORB algorithm to suit our VINS-MKF. We preprocessed multiple camera images and combined multiple images as an input image of feature extraction, as we found in the experiment that using a merged image could reduce the time-consuming of building scale pyramid on the GPU, and this could maximum efficiency with the GPU. This is different from the original ORB algorithm, the result of our feature extraction is multiple images’ feature information instead of a single image’s. 

### 4.3. Tracking

**Figure 6:**
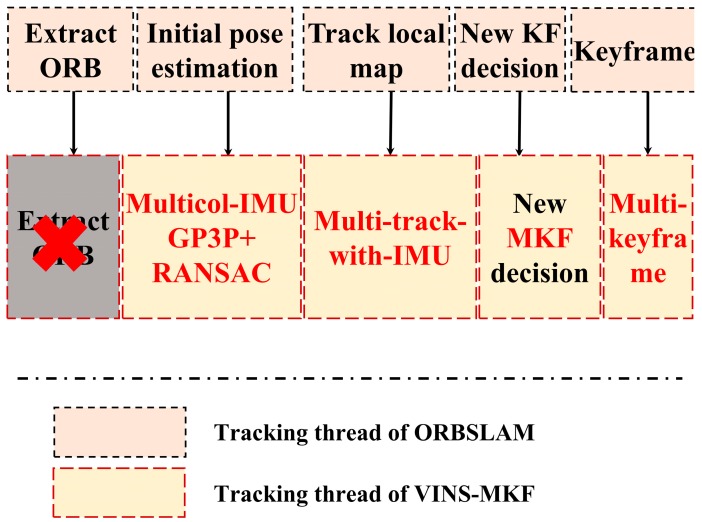
The comparison between the tracking procedure of VINS-MKF and ORBSLAM. The procedure of our proposed VINS-MKF is similar to ORBSLAM, while several adjustments were adopted in the steps.

The tracking thread is the core of the proposed VINS-MKF system, its functions and procedure are similar to ORBSLAM. As for the tracking functions, the tracking thread localizes the multi-camera pose by handing the current multi-frame and the tracking thread decides to select and to spawn a new MKF. Figure 6 compares the tracking procedure of the VINS-MKF and the ORBSLAM. The profound improvements of VINS-MKF over the original ORBSLAM in the tracking procedure can be summarized as the introduction of MKF and the tightly coupled IMU information, along with the introduction of hyper graph, a different initial pose estimation method with multi-frame, the different co-visibility graph, and motion-only BA (Bundle Adjustment) optimization, and the additional criterion for spawning a new MKF.

#### 4.3.1. The Introduction of the Hyper Graph

**Figure 7:**
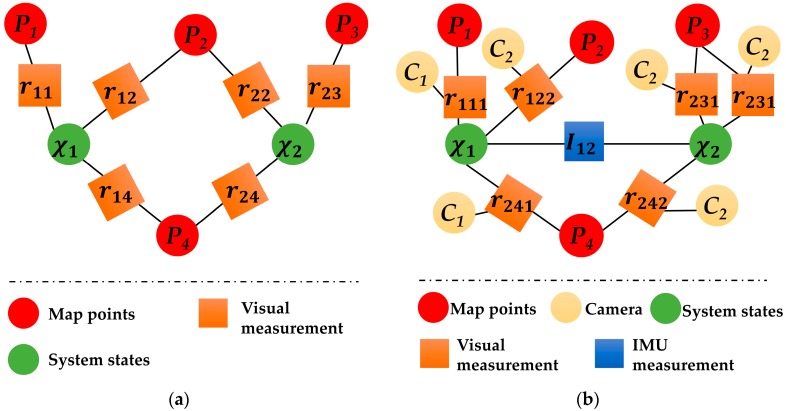
The different factor graphs. (**a**) factor graph of common visual odometry (VO) method; (**b**) factor graph of the proposed VINS-MKF.

Compared to ORBSLAM, an important modification of the proposed VINS-MKF is that we used the hyper graph to model the tracking and local mapping pipeline, as Figure 7b shows. Besides, the introduction of the MultiCol-IMU model and the tightly coupled IMU information made the tracking more accurate and robust. 

#### 4.3.2. Different Initial MF Pose Prediction Method

Compared to ORBSLAM, we introduced the multi-track-with-IMU model for initial pose prediction, and we introduced an improved re-localization method for initial MF pose prediction.

**Figure 8:**
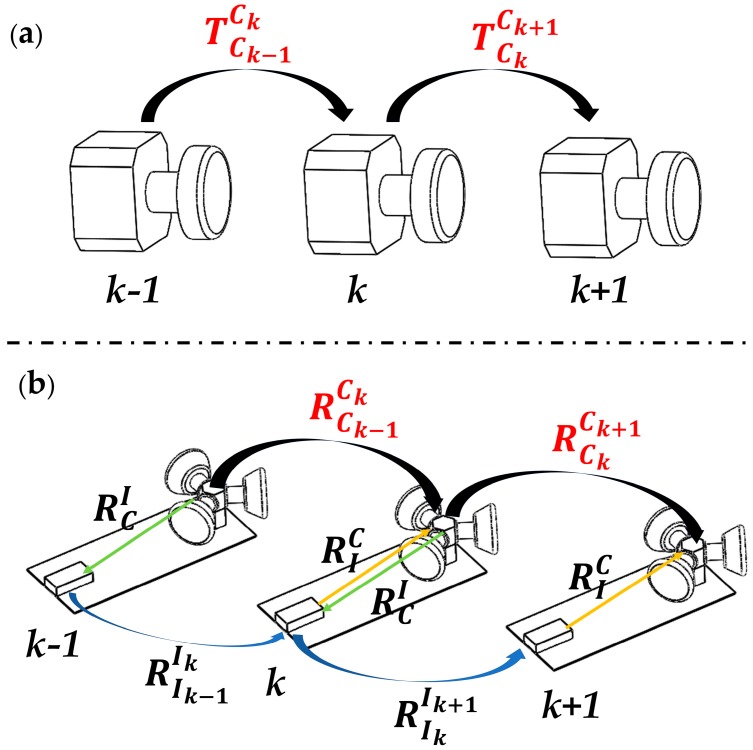
Different pose prediction model. (**a**) Constant velocity model; (**b**) multi-track-with-IMU model. In ORBSLAM, the camera pose predicted by the constant velocity motion model, in the proposed VINS-MKF, the multi-track-with-IMU model is used to predict the initial MF pose.

For the initial MF pose estimation, there two different situations:

**When the tracking was successful**, the initial MF pose was predicted by the proposed multi-track-with-IMU model in Figure 8b, which bridged the transition between two consecutive multi-frames and supplied an optimum initial value. The constant velocity motion model used in ORBSLAM turns out to be suboptimal when it comes to higher motion dynamics, and the gained less accurate initial value resulted in higher convergence periods, or in the worst case, the optimization did not converge at all. 

**When the tracking failed**, an improved relocalization method was used to estimate the initial MF pose, which combined GP3P [52] and RANSAC, with the map points being assigned to a set of recent MKFs. This was different from ORB-SLAM, where a single camera and non-minimal PnP solver is used in relocalization.

#### 4.3.3. The Different Co-Visibility Graph and the Motion-Only BA Optimization Method

**Figure 9:**
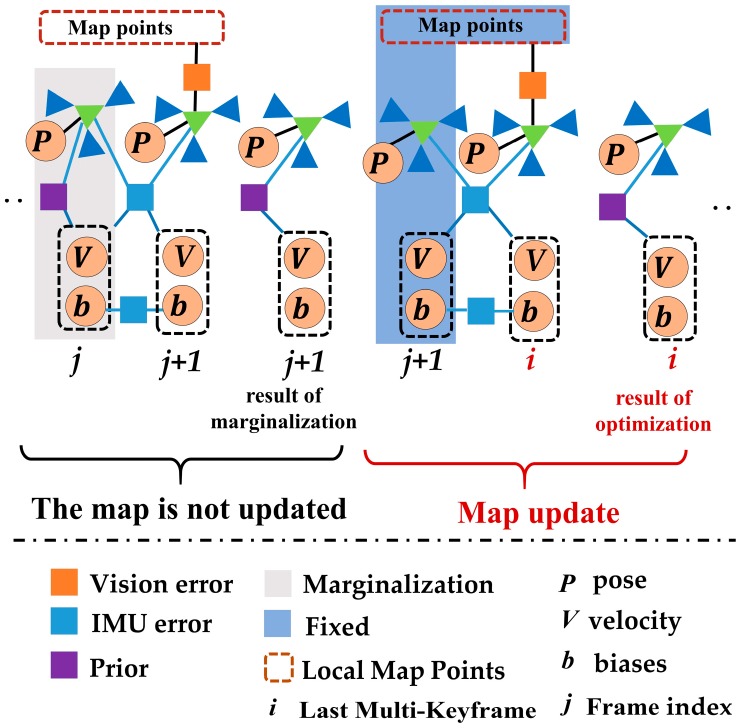
Motion-only BA optimization for tracking local map in our VINS-MKF.

In the proposed VINS-MKF, both the local map based on the co-visibility graph and the motion-only BA optimization for tracking local map contained a difference with ORBSLAM. The co-visibility graph that used to build a local map in the VINS-MKF contained multi-frames instead of the single frames. The most important improvement was the motion-only BA optimization for tracking the local map. Compared to ORBSLAM, we optimized the current multi-frame by minimizing the feature reprojection error of all matched points and an IMU error term. It has two optimization models depending on the map update or not (by the Local Mapping), as illustrated in Figure 9. Inspired by Reference [24], we performed the following two motion-only BA optimization nonlinear optimizations:

- Assuming no map update, we performed the nonlinear optimization as Equation (11).
  $$\chi^{*}=\mathrm{argmin}\;\left\{\|e_{prior}{\|}_{\Sigma_{0}}^{2}+\sum_{t}\|e{\left(Z_{It}^{t+1})\right\|}_{\sum_{t}^{t+1}}^{2}+\sum_{c=1,2,3}\sum_{t}\sum_{s}H\left(\|e{\left(Z_{stc})\right\|}_{\Sigma_{stc}}^{2}\right)\right\}.$$
- Assuming that the map updated, the nonlinear optimization in Equation (11) changed to: F
  (21) $$\chi^{*}=\mathrm{argmin}\;\left\{\sum_{t}\|e{\left(Z_{It}^{t+1})\right\|}_{\Sigma_{t}^{t+1}}^{2}+\sum_{c=1,2,3}\sum_{t}\sum_{s}H\left(\|e{\left(Z_{stc})\right\|}_{\Sigma_{stc}}^{2}\right)\right\}.$$

#### 4.3.4. Additional Criterion for Spawning a New MKF 

For the proposed VINS-MKF, the tracking thread decided when to spawn a new MKF to the local mapping thread. As a 360^◦^ view of the environment was present at all time, the reconstruction quality was suffered by the vast inserted MKFs, thus we added an additional criterion: A minimum distance between the current MCS pose and the reference must be exceeded. This value can be estimated by the median scene depth.

### 4.4. Local Mapping

**Figure 10:**
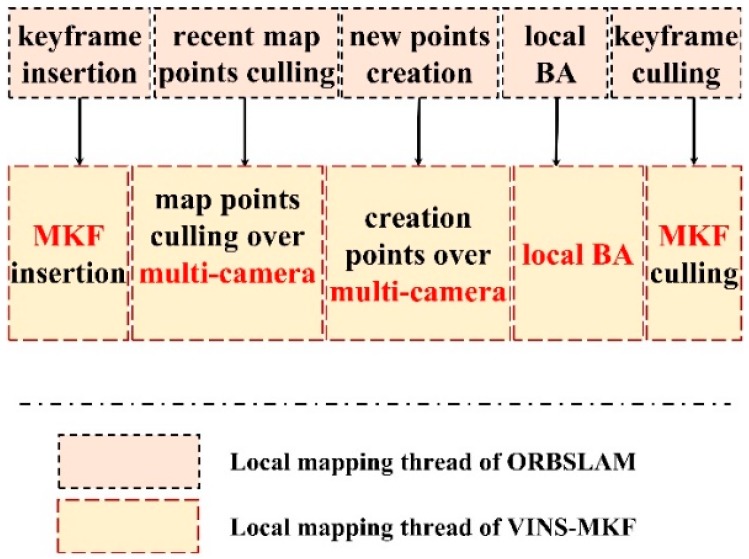
The difference between VINS-MKF and ORBSLAM with regard to the local mapping thread. For VINS-MKF, once the tracking thread spawns a new MKF into the map, the mapping thread updates the co-visibility graph and saves the new MKF’s Bag-of-Words representation. Local mapping also creates new map points between the new MKF and its connected MKFs, removes the outlier map points and MKFs.

In our proposed VINS-MKF, once a new MKF was spawned in the tracking thread, the local mapping began to process the new MKF and perform optimization to achieve an optimal reconstruction. The procedure of local mapping and its difference with ORBSLAM are shown in Figure 10. The two profound adjustments between VINS-MKF and ORBSLAM are the structure of the double window and the criterions for MKF deletion.

#### 4.4.1. Improved Double Window Structure

**Figure 11:**
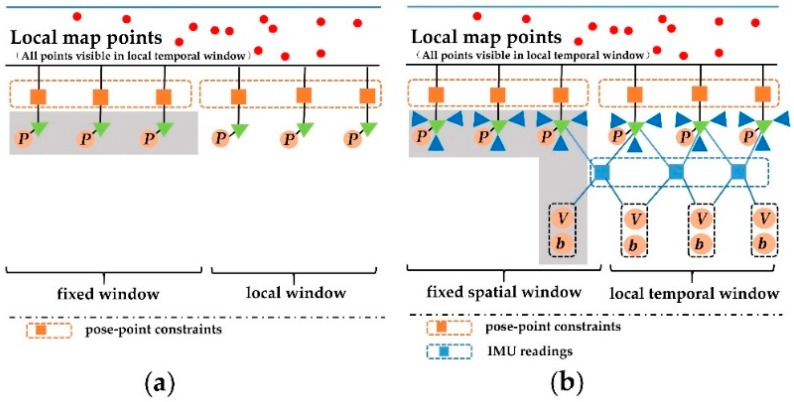
The comparison of the double window structure. (**a**) The double window structure of ORBSLAM; (**b**) the improved temporal-spatial double window structure of VINS-MKF.

An improved double window structure [24,35] was built in our VINS-MKF, which was retrieved from a local visible map, to organize variables to be optimized and related observations, as Figure 11b shows. The local temporal window included the last T MKFs (The MKF T +1 was always included in the fixed spatial window as it constrained the IMU states), which was related by pose-point constraints and the IMU readings. The fixed spatial window included S MKFs, which were not in the local temporal window, but shared observations of the local points. The MKFs in the fixed spatial window remained fixed during the optimization, but they contributed to the total cost. The improved double window also included map points that were observed by at least two MKFs. The difference between our double window structure and ORBSLAM can be found in Figure 11.

#### 4.4.2. Different MKF Deletion Criteria 

The criteria for MKF deletion in the VINS-MKF were different from the original ORBSLAM. We could not discard MKFs arbitrarily, as the IMU information constrained the motion of consecutive MKFs. The redundant MKFs were allowed to be deleted only in the following two conditions:

- The two consecutive MKFs in the local temporal window differ by more than 0.5 s, the reason is that the longer the temporal difference between consecutive MKFs, the weaker the information IMU provides.
- Any two consecutive MKFs differing less than 3 s, the reason is that we needed to perform full BA and to refine a map at any time. If we switched off full BA with IMU constraints, we would only need to restrict the temporal offset between keyframes in the local spatial window.

## 5. Experiments

We performed various experiments to evaluate the performance of the proposed VINS-MKF, and those experiments were performed on our home-made datasets and the real corridor environment. In the first experiment, we performed several comparison analyses to show the benefits of using multiple fisheye cameras. In the second experiment, we demonstrated the advantages of tightly coupling the IMU in the VINS-MKF. For the third experiment, we evaluated the efficiency of the proposed VINS-MKF system. Finally, Experiment 4 validated the capabilities of our VINS-MKF system in comparison to the state-of-art VINS-Mono algorithm. 

**Experimental setup**: We applied our algorithm on our home-made handheld multi-camera visual inertial platform, as Figure 4b shows. The platform was equipped with a Microstrain 3DM-GX3 IMU (LORD Microstrain, Williston, VT, USA) and three mvBlueFOX-MLC200w grayscale cameras sensors (Matrix Vision Gmbh, Oppenweiler, Germany) with 185°Lensagon BF2M12520 lens (Lensation GmbH, Karlsruhe, Germany). Three cameras were distributed on the three sides of an equilateral triangle platform, providing a 360-degree annular viewing angle, and they capture 752 × 480 images at 20 Hz. The Microstrain 3DM-GX3 IMU runs at 200 Hz. The calibration of the camera system and the camera-IMU system were performed previously. All experiments were performed on an Intel Core i5-6300HQ CPU @2.30 GHz (Intel Corporation, Santa Clara, CA, USA) laptop computer with 12 GB RAM. Besides, to measure the ground truth data of the multi-camera visual inertial platform pose on the home-made datasets, in this work, we used an external Optitrack tracking system, which comprised eight infrared cameras. After attaching several highly reflective markers to the platform, the tracking system could provide six DOF pose estimates of the platform with a frequency of up to 200 fps.

**Figure 12:**
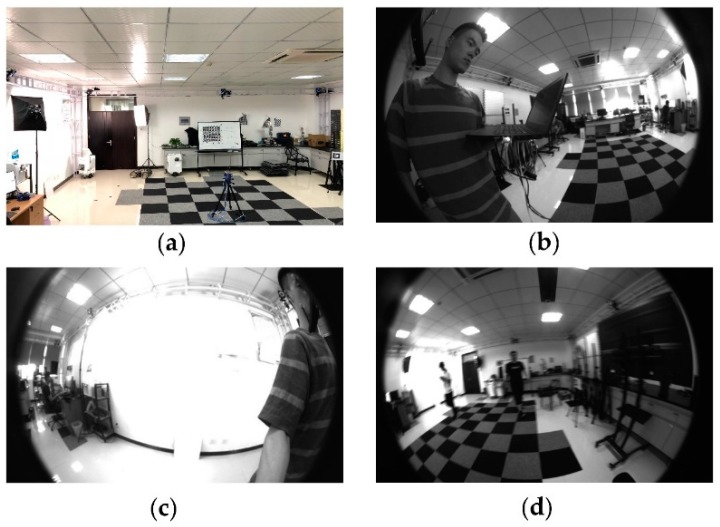
The experimental and datasets environment. (**a**) Indoor environment; (**b**) the normal condition in datasets; (**c**) the texture-less area and over exposure condition; (**d**) the pedestrians and aggressive motion.

**Home-made Datasets:** As far as we know, there no datasets include both multiple fisheye cameras and IMU information that were suitable for our work, so we evaluated the proposed VINS-MKF algorithm on our home-made indoor datasets, include static and dynamic datasets. The experimental and datasets environment is as Figure 12 shows. We chose our indoor laboratory environment as the experiment area, which had a size of 5 m × 5 m, as Figure 12a shows. We used a hand-held platform to move into the room, and we recorded data from all synchronized cameras and the IMU, the obstacles we laid in the room were randomly deployed. Multiple sequences in those datasets were recorded with various conditions, comprising two different walking shapes, texture-less area, over exposure, pedestrians, and aggressive motion. The items in those datasets and the difference between them are shown in Table 1. The home-made datasets provide six datasets/trajectories in total. Each trajectory contains camera images with 752 × 480 resolution from synchronized multiple fisheye cameras, the synchronized IMU measurements also included. Besides, each trajectory also includes an extrinsic and intrinsic calibration of the sensors, as well as ground truth trajectories that were obtained using the external motion tracking system. Note that although the experiments were done offboard on the PC, they are done in real-time, i.e., the video streams are asynchronously replayed at the original frame rate of the cameras.

### 5.1. Experiment 1: Impact of Multiple Cameras 

The purpose of this experiment was to demonstrate the benefits of using multiple fisheye cameras. We compared three different camera configurations on both home-made datasets and the corridor environment. To simplify the notation, we used VO-MKF, which stands for the VINS-MKF without fusing the IMU information, VO-Mono was the VINS-MKF with a monocular camera (in this paper, we selected camera 0) and without an IMU, and VO-Stereo was defined as VINS-MKF with two arbitrary cameras and without an IMU.

#### 5.1.1. Experiment 1 on Home-Made Datasets

**Figure 13:**
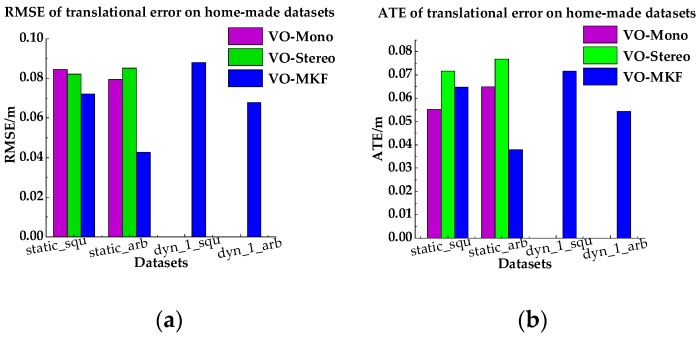
RMSEs and Median ATEs for VO-Mono, VO-Stereo and VO-MKF. (**a**) RMSEs of translational error; (**b**) median ATEs of translation.

**Figure 14:**
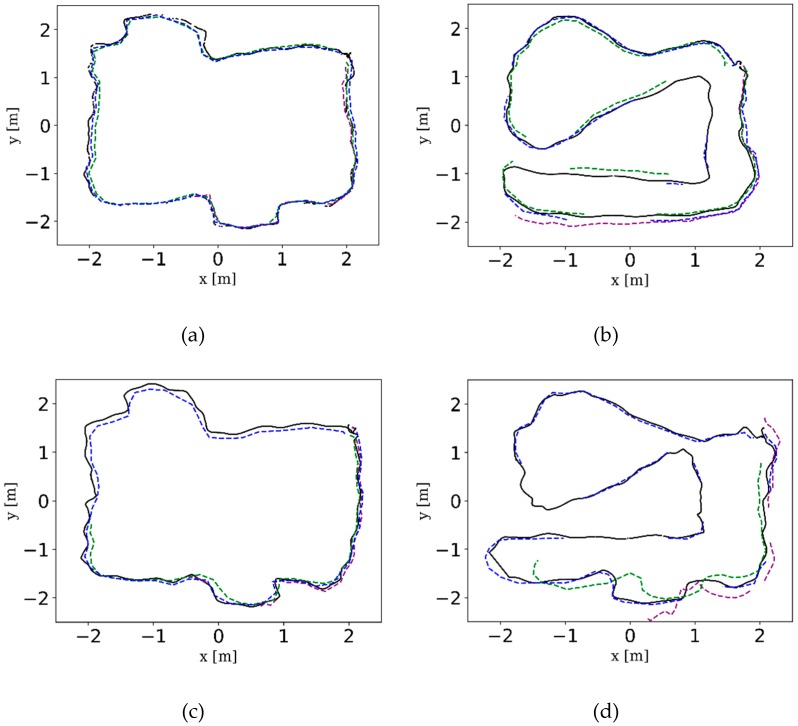
The comparison trajectories of VO-Mono (Purple dotted line), VO-Stereo (Green dotted line), VO-MKF (Blue dotted line), and the ground truth (Black solid line) as provided by Optitrack tracking system on different datasets. (**a**) static_squ; (**b**) static_arb; (**c**) dyn_1_squ; (**d**) dyn_1_arb. It should be noted that some trajectories in the above figures looked intermittent, such as between point A and B in Figure 14b; however, this does not mean the method concerned fails to run in the sequence, the trajectories actually exist. The reason for this case is due to the MKF culling mechanism described in Section 4.4.2. Since our indoor environment was not big enough, co-visibility between MKFs was existent, so that the MKFs are deleted during the trajectory, but the drawing software only plots the recorded MKFs, thus it seems that tracking is lost.

In this experiment, we compare VO-MKF, VO-Mono and VO-Stereo on the static and dyn_1 datasets. To demonstrate the results of comparisons quantitatively, we used the root mean square error (RMSE) and absolute trajectory error (ATE) as the evaluation metrics, as described in the appendix. Table 2 shows the corresponding RMSE and ATE results, and Figure 13 are tuitively histograms of Table 2. Comparing Table 2 and Figure 13, we found that VO-MKF shows the best accuracy on all the datasets and the lowest RMSE and ATE values than VO-Mono and VO-Stereo. The reason is that the large FOV of VO-MKF provides redundant information and makes up for the texture-less area and over exposure conditions in the datasets, but those conditions are difficult for VO-Mono and VO-Stereo. Notice that although we recorded part of the results of VO-Mono, it failed on both the static datasets, as can be seen in Figure 14a.

The intuitive trajectories estimated by the three camera configurations and the ground truth trajectories provided by Optitrack tracking system are shown in Figure 14. It can be clearly seen that VO-MKF gained the least amount of errors and its trajectories had the best alignment with the ground truth in all datasets than VO-Mono and VO-Stereo. The VO-Mono (Purple dotted line) had poor robustness to the texture-less area and overexposure conditions, and its data points are break off in all the experiments. The data in Figure 14c,d shows that VO-Stereo was more sensitive to the blur images caused by moving pedestrians and failed in both the dynamic datasets, while VO-MKF performed better during the whole experiments.

In summary, the above results demonstrated that VO-MKF configurated with multiple cameras did provide the best accuracy and robustness in terms of the state estimation than VO-Mono and VO-Stereo.

#### 5.1.2. Experiment 1 on Corridor Environment

**Figure 15:**
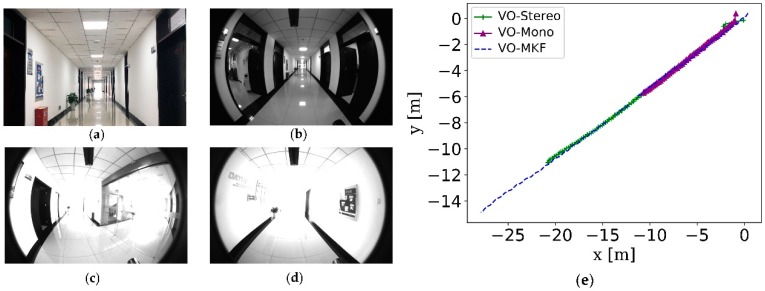
The corridor environment and the comparison trajectories of VO-Mono, VO-Stereo and VO-MKF in corridor environment. The difficult texture-less corridor environment is shown in (**a**,**b**), the challenging overexposure condition is shown in (**c**,**d**). The comparison trajectories are shown in (**e**).

In this part, we mainly validated the robustness of VO-MKF in a corridor environment (Figure 15a–d), since it was a challenging environment for visual state estimation systems. Comparing the resulted trajectories in Figure 15e, we can see that VO-MKF had the best robust performance, both VO-Mono and VO-Stereo failed during the experiment, since the texture-less and changing illumination conditions in corridor were too difficult for the limited visual FOV.

### 5.2. Experiment 2: Impact of Tightly-Coupled IMU

The goal of this experiment was to demonstrate the benefits of tightly coupling the IMU measurements. In the Experiment 1, we found that VO-MKF had a poor performance in the dynamic datasets than in the static datasets, as we could analyze from Figure 13, besides, VO-MKF had a lower robustness under the conditions in Figure 15d. Thus, we compared the performance of VO-MKF and the VINS-MKF on dyn_1, dyn_2, and corridor environment, with respect to the changes in the texture-less area, over exposure conditions and pedestrian motion. 

**Figure 16:**
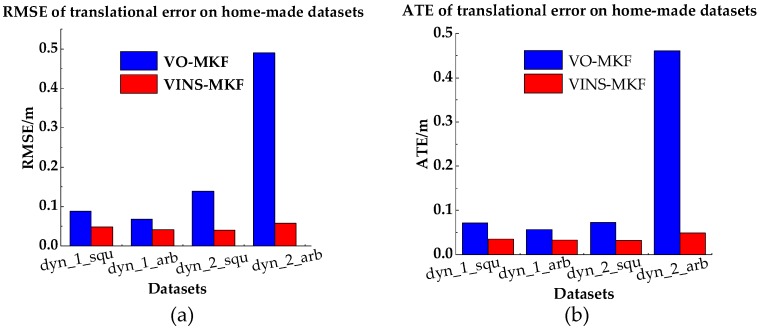
RMSEs and Median ATEs for VINS-MKF, VO-MKF. (**a**) RMSEs of translational error; (**b**) Median ATEs of translation.

**Figure 17:**
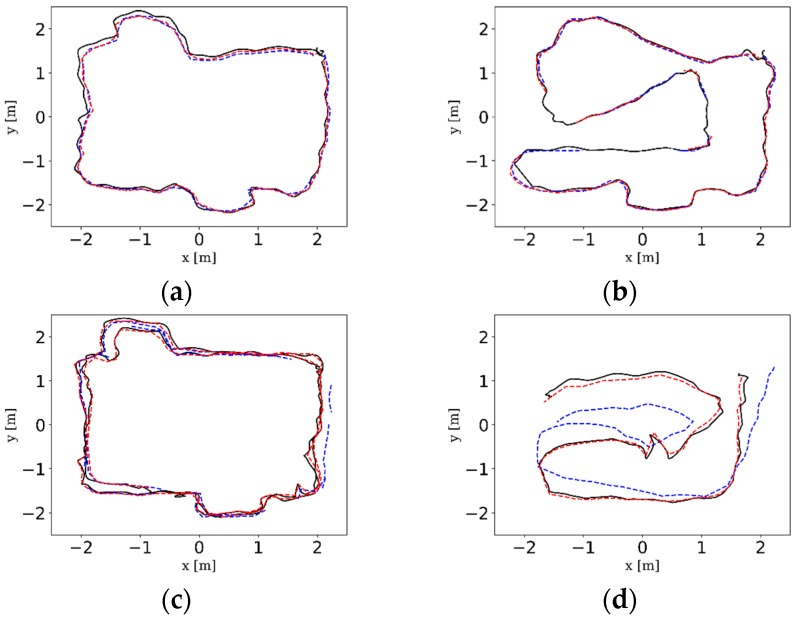
The estimated trajectories of VINS-MKF (Red dotted line), VO-MKF (Blue dotted line) and the ground truth (Black solid line) on different datasets. (**a**) dyn_1_squ; (**b**) dyn_1_arb; (**c**) dyn_2_static; (**d**) dyn_2_arb.

**Figure 18:**
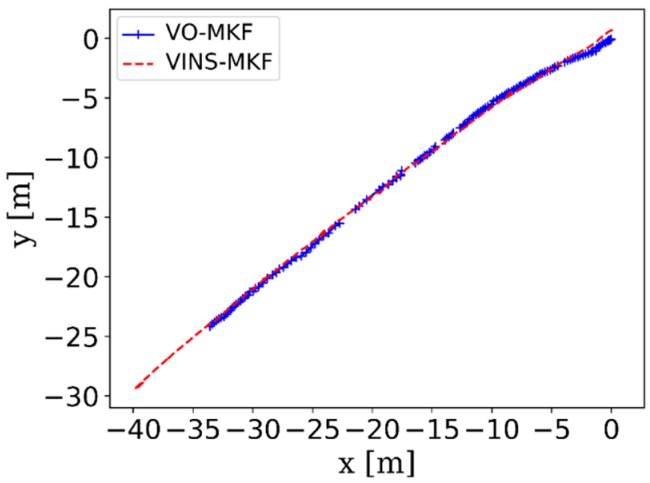
The trajectories of VINS-MKF (Red) and VO-MKF (Blue) in corridor environment.

The comparison RMSE results and ATE results are shown in Table 3 and Figure 16. It can be seen that the VINS-MKF shows lower RMSEs and ATEs than VO-MKF in dyn_1 datasets. The reason is that the inertial measurements in the VINS-MKF can make up for the low-quality visual information caused by the difficult conditions on datasets. The advantage of VINS-MKF was more obvious than VO-MKF, in the dyn_2 datasets, which has extra rapid motion condition. The superior performance of the VINS-MKF could also be demonstrated from the trajectories in Figure 17, where the VINS-MKF trajectories had better alignment with the ground truth than VO-MKF, especially in Figure 17c. One explanation is that VO-MKF was less resistant to the aggressive motion in dyn_2 datasets. The robustness of the VINS-MKF was also verified in the corridor environment, as we can see from Figure 18, VINS-MKF (Red line) has obvious long-term stability than VO-MKF (Blue line). 

These results indicated that the VINS-MKF performs better than VO-MKF and the tightly-coupled IMU information benefitted the state estimation. 

### 5.3. Efficiency Evaluation

**Figure 19:**
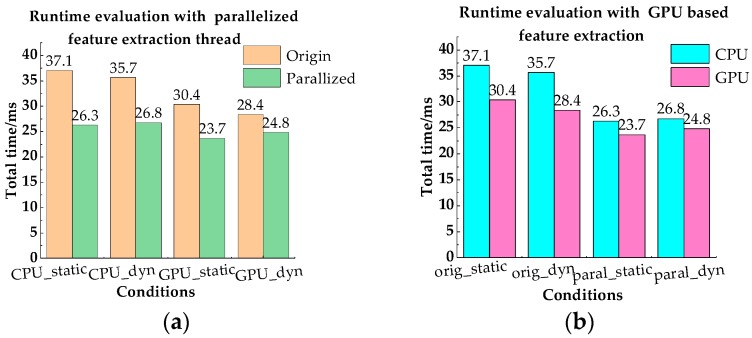
Runtime evaluation with the parallelized feature extraction thread and the GPU based feature extraction.

In this part, we validated the acceleration effect of GPU based feature extraction and the parallelized feature extraction thread under four different feature extraction conditions. It was noted that this experiment was performed on an Intel Core i7-4710MQ CPU @2.50 GHz desktop PC with 16 GB RAM. Table 4 and Table 5 show the evaluated execution time of each condition in the static and dynamic dataset respectively, and it can be seen that the “Parallel_GPU” condition had the lowest feature extraction time and total time on both datasets. The reason can be summarized from Figure 19. From Figure 19a,b, we can see that the use of GPU in feature extraction speedup, both in feature extraction and the total VINS-MKF. The parallelized feature extraction strategy significantly improved the efficiency of VINS-MKF, as can be seen from Figure 19b, but the parallel strategy had little effect on feature extraction computational efficiency.

### 5.4. Comparison between the Proposed Vins-MKF and Vins-Mono

In this experiment, we validated the advantages of the proposed VINS-MKF by comparing against the VINS-Mono [25], which is a representative state-of-the-art tightly-coupled visual-inertial state estimation method and has open source implementation.

**Figure 20:**
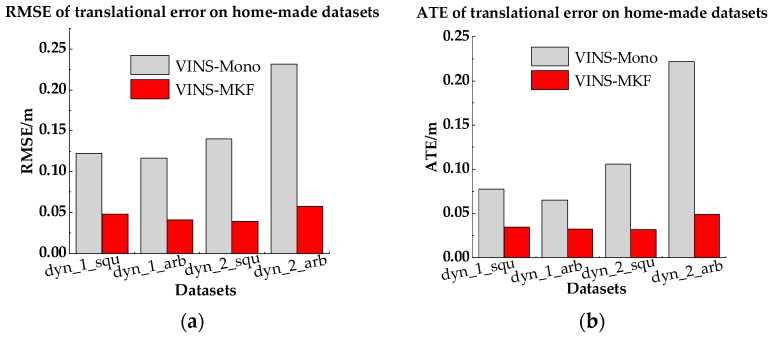
RMSEs and Median ATEs for VINS-MKF, VINS-Mono. (**a**) RMSEs of translational error; (**b**) Median ATEs of translation.

**Figure 21:**
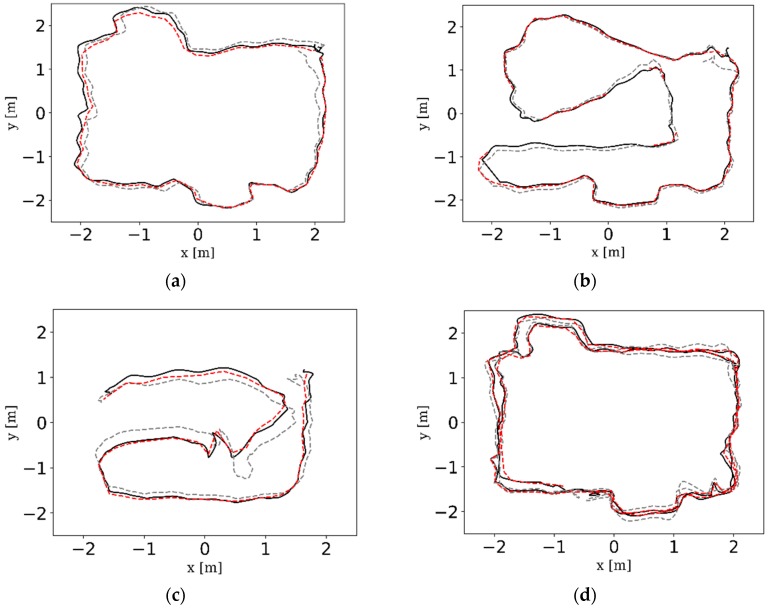
The estimated trajectories of VINS-MKF (Red dotted line), VINS-Mono (Gray dotted line) and the ground truth (Black solid line) on different datasets. (**a**) dyn_1_squ; (**b**) dyn_1_arb; (**c**) dyn_2_static; (**d**) dyn_2_arb.

The comparison results are presented in Table 6, Figure 20 and Figure 21. From the intuitive histograms in Figure 20, we can directly see that the VINS-MKF significantly outperformed VINS-Mono [25] and had lower RMSEs and ATEs. The trajectories estimated by those two methods are plotted in Figure 21, from which we can clear see that the VINS-MKF trajectories fit well with the ground truth, while VINS-Mono [25] gained many more drifts, especially in the dynamic_2 dataset. The reasons for this may be as followings: First, the VINS-Mono’s marginalization mechanism that ignores the previous observation results in the drifts. Besides, the limited FOV of VINS-Mono [25] cannot provide complementary information when it comes to extreme texture-less, overexposure, pedestrians, and aggressive motions in our datasets, while our VINS-MKF benefits from the abundant multi-camera visual-inertial information, and the variable management mechanism, etc.

## 6. Conclusions and Future Work

In this paper, we presented the novel tightly-coupled multi-keyframe visual-inertial odometry algorithm VINS-MKF, which ensured accurate and robust state estimation for robots in an indoor environment. The performance of proposed VINS-MKF benefited from the efficient and vast information provided by the multi-camera and IMU, it also benefited from the formulated nonlinear optimization method. Furthermore, the proposed VINS-MKF addressed the efficiency problem by proposing a novel framework, in which the feature extraction, tracking and mapping were parallelized. The tightly-coupled multi-keyframe visual-inertial nonlinear optimization also ensured the accuracy of VINS-MKF. The proposed novel state estimation framework of VINS-MKF greatly improves the efficiency of state estimation, which includes three parallelized thread: GPU based feature extraction, tracking and local mapping. Furthermore, the state estimation for the VINS-MKF is attractive for its novel MutiCol-IMU camera model, a hyper-graph-based state estimation structure. We verify the performance of the proposed VINS-MKF through various comparison experiments, including a comparison against the state-of-art VINS-Mono [25] algorithm. Future work will mainly focus on a more accurate and general initialization method, a robust state estimation model for a dynamic indoor environment, and the dense mapping for local obstacle avoidance.

## Figures and Tables

**Table 1:** Home-made datasets and the respective included items.

Datasets	Shape	Texture-Less	Over Exposure	Pedestrians	Aggressive Motion
static	squ ^1^	√	√		
	arb ^1^	√	√		
dyn ^1^_1	squ	√	√	√	
	arb	√	√	√	
dyn_2	squ	√	√	√	√
	arb	√	√	√	√

^1^ dyn, squ and arb are the abbreviation of dynamic, square, and arbitrary, respectively. The square shape means that we walk along the shape of carpet in Figure 12a, and the arbitrary shape means that we walk arbitrarily in the room.

**Table 2:** The root mean square error (RMSE/m) and absolute trajectory error (ATE/m) results of VO-Mono, VO-Stereo and VO-MKF on home-made datasets.

Datasets	Algorithms	RMSE/GT Scale	ATE		
			Mean	Median	Std
static_squ	VO-Mono	0.084	0.068	0.055	0.050
	VO-Stereo	0.082	0.0736	0.072	0.038
	VO-MKF	0.072	0.066	0.065	0.029
static_arb	VO-Mono	0.079	0.067	0.065	0.043
	VO-Stereo	0.085	0.081	0.077	0.026
	VO-MKF	0.043	0.040	0.038	0.014
dyn_1_squ	VO-Mono	×	×	×	×
	VO-Stereo	×	×	×	×
	VO-MKF	0.088	0.081	0.072	0.035
dyn_1_arb	VO-Mono	×	×	×	×
	VO-Stereo	×	×	×	×
	VO-MKF	0.068	0.061	0.054	0.029

**Table 3:** The root mean square error (RMSE/m) and absolute trajectory error (ATE/m) results of VO-MKF and VINS-MKF on dynamic datasets.

Datasets	Algorithms	RMSE/GT Scale	ATE		
			Mean	Median	Std
dyn_1_squ	VO-MKF	0.088	0.081	0.072	0.035
	VINS-MKF	0.048	0.041	0.035	0.026
dyn_1_arb	VO-MKF	0.068	0.062	0.057	0.029
	VINS-MKF	0.041	0.034	0.032	0.022
dyn_2_squ	VO-MKF VINS-MKF	0.138 0.0395	0.107 0.035	0.072 0.032	0.087 0.018
dyn_2_arb	VO-MKF	0.490	0.433	0.460	0.228
	VINS-MKF	0.057	0.052	0.049	0.024

**Table 4:** Runtime (ms) performance of VINS-MKF with different feature extraction conditions on static dataset.

Conditions	Feature Extraction	Tracking	Mapping	Total Time	
Origin ^2^_CPU ^2^	18.2	17.3	31.3	37.1	
Parallel ^2^_CPU	18.6	19.7	31	26.3	
Origin_GPU ^2^	11	17.7	30.3	30.4	
Parallel_GPU	11.1	17.6	30.7	23.7	

^2^ The “Origin “means feature extraction isn’t parallelized, “CPU” means feature extraction without GPU acceleration, “Parallel” means feature extraction is parallelized, and “GPU” means feature extraction is accelerated by GPU.

**Table 5:** Runtime(ms) performance of VINS-MKF with different feature extraction conditions on dynamic dataset.

Conditions	Feature Extraction	Tracking	Mapping	Total Time
Origin_CPU	18.6	15.4	39.4	35.7
Parallel_CPU	18.9	20	38.2	26.8
Origin_GPU	10.9	15.9	38.9	28.4
Parallel_GPU	11.1	18.2	37.6	24.8

**Table 6:** The root mean square error (RMSE/m) and absolute trajectory error (ATE/m) results of VINS-Mono and VINS-MKF on home-made datasets.

Datasets	Algorithms	RMSE/GT Scale	ATE		
			Mean	Median	Std
dyn_1_squ	VINS-Mono	0.122	0.100	0.077	0.071
	VINS-MKF	0.048	0.041	0.035	0.026
dyn_1_arb	VINS-Mono	0.116	0.094	0.065	0.070
	VINS-MKF	0.041	0.034	0.032	0.022
dyn_2_squ	VINS-Mono	0.149	0.115	0.106	0.080
	VINS-MKF	0.039	0.035	0.032	0.018
dyn_2_arb	VINS-Mono	0.232	0.210	0.222	0.106
	VINS-MKF	0.057	0.052	0.049	0.024

## Appendix A

The calculation formulas and definition of RMSE and ATE are as following:

Assuming both the sequences’ length of the estimated trajectory and the ground truth trajectory is *n*, $P_{t}$ are the estimated camera poses, $P_{t}^{g}$ are the ground truth poses. We can use the two metrics RMSE and/or ATE to compare $P_{t}$ and $P_{t}^{g}$ at time *t*. The relative pose error between $P_{t}$ and $P_{t}^{g}$ can be expressed by the relative orientation $P_{t}^{ro}$:

(A1) $$P_{t}^{ro}=P_{t}^{g-1}P_{t}\;$$

In order to calculate the absolute error, the two trajectories need to be aligned in advance using a similarity transformation *S*. For *n* pose pairs, RMSE is the root mean square error over the translational differences between the two trajectories:

(A2) $$\;\mathrm{RMSE}={\left(\frac{1}{n}\sum_{t=1}^{n}\|\mathrm{trans}{\left(P_{t}^{ro})\right\|}^{2}\right)}^{\frac{1}{2}}={\left(\frac{1}{n}\sum_{t=1}^{n}\|\mathrm{trans}{\left(P_{t}^{g-1}S\;P_{t})\right\|}^{2}\right)}^{\frac{1}{2}}\;$$

“trans” is the translational component of the transformation matrix M.

ATE is the absolute trajectory error, we define three calculation ways for ATE:

(A3) $$\mathrm{ATE}\;(\mathrm{mean})=\;\frac{1}{n}\sum_{t=1}^{n}\|\mathrm{trans}\left(P_{t}^{ro}\right)\|=\frac{1}{n}\sum_{t=1}^{n}\|\mathrm{trans}\left(P_{t}^{g-1}S\;P_{t})\right\|$$

(A4) $$\mathrm{ATE}\;(\mathrm{median})=\;\mathrm{median}\;(\|\mathrm{trans}\left(P_{t}^{ro})\right\|)=\mathrm{median}\;(\|\mathrm{trans}\left(P_{t}^{g-1}S\;P_{t})\|\right),\;t\in \left(1,n\right)\;$$

(A5) $$\mathrm{ATE}\;(\mathrm{std})={\left(\frac{1}{n}\sum_{t=1}^{n}\|\mathrm{trans}\left(P_{t}^{ro}\right)-\mathrm{ATE}{\left(\mathrm{mean})\right\|}^{2}\right)}^{\frac{1}{2}}\;$$

It should be noted that RMSE can be seen as one calculation way of ATE, as many other works do [18,53]. In this paper, since the RMSE attributes more influence to outliers, we distinguish RMSE with other ATE ways.
